# A community focused approach toward making healthy and affordable daily diet recommendations

**DOI:** 10.3389/fdata.2023.1086212

**Published:** 2023-11-06

**Authors:** Joe Germino, Annalisa Szymanski, Heather A. Eicher-Miller, Ronald Metoyer, Nitesh V. Chawla

**Affiliations:** ^1^Department of Computer Science and Engineering, Lucy Family Institute, University of Notre Dame, Notre Dame, IN, United States; ^2^Department of Nutrition Science, Purdue University, West Lafayette, IN, United States

**Keywords:** linear programming, food recommendation, diet cost, community-centered optimization, food information networks, nutrition

## Abstract

**Introduction:**

Maintaining an affordable and nutritious diet can be challenging, especially for those living under the conditions of poverty. To fulfill a healthy diet, consumers must make difficult decisions within a complicated food landscape. Decisions must factor information on health and budget constraints, the food supply and pricing options at local grocery stores, and nutrition and portion guidelines provided by government services. Information to support food choice decisions is often inconsistent and challenging to find, making it difficult for consumers to make informed, optimal decisions. This is especially true for low-income and Supplemental Nutrition Assistance Program (SNAP) households which have additional time and cost constraints that impact their food purchases and ultimately leave them more susceptible to malnutrition and obesity. The goal of this paper is to demonstrate how the integration of data from local grocery stores and federal government databases can be used to assist specific communities in meeting their unique health and budget challenges.

**Methods:**

We discuss many of the challenges of integrating multiple data sources, such as inconsistent data availability and misleading nutrition labels. We conduct a case study using linear programming to identify a healthy meal plan that stays within a limited SNAP budget and also adheres to the Dietary Guidelines for Americans. Finally, we explore the main drivers of cost of local food products with emphasis on the nutrients determined by the USDA as areas of focus: added sugars, saturated fat, and sodium.

**Results and discussion:**

Our case study results suggest that such an optimization model can be used to facilitate food purchasing decisions within a given community. By focusing on the community level, our results will inform future work navigating the complex networks of food information to build global recommendation systems.

## 1. Introduction

Research has shown that while many Americans pay attention to healthy eating habits, they are often burdened with obstacles that limit their finding of food choices that are both nutritious and affordable (Funk and Kennedy, 2016; Zorbas et al., 2018). This is especially true for residents living in conditions of poverty who are not only often isolated from access to quality food, but are also limited in economic stability and nutrition education to make the most informed food choices for their health needs (Zorbas et al., 2018; Allcott et al., 2019; De Leon et al., 2020). Because of these obstacles, many individuals in poverty struggle with food insecurity, and as a result, suffer higher risks of chronic health diseases, such as malnutrition and obesity (Treuhaft and Karpyn, 2010; Hanson and Connor, 2014; Leung et al., 2014; Gregory and Coleman-Jensen, 2017; Hartline-Grafton and Dean, 2017). To assist low-income households, the Supplemental Nutrition Assistance Program (SNAP), a federal supplementary aid program, provides monthly benefits up to $250 for single person households (Food and Nutrition Service U.S. Department of Agriculture, 2021). Although SNAP has mitigated food insecurity by 30%, many SNAP families are still unable to afford nutritious foods that fulfill the US Department of Agriculture's (USDA) Dietary Guidelines for Americans (DGA) for healthy eating (Mulik and Haynes-Maslow, 2017). A change in food choice behavior toward nutritious well-balanced diets that are affordable is crucial in reducing the health risks of these households (Ruder et al., 2022).

The ability to balance nutritional goals with the competing constraints of time and money make it difficult for SNAP consumers to make healthy food choices (Mancino and Guthrie, 2014). To achieve a healthy diet, consumers must be equipped to identify foods that are nutritious based on their dietary needs and also determine whether they are locally available and affordable. Consumers must have the ability to understand the nutritional value of foods and how much daily intake of these food products would fulfill federal dietary guidelines outlined by government services, and whether or not the costs fit their limited budgets. Current recommendation systems pose complications because they do not account for affordable pricing nor the lack of availability of specific food products that some communities face. There is a need to create a community-centered recommendation model that focuses on the products available at local grocery stores to assist SNAP participants with food purchase decisions that combine nutrition and cost information to find an optimal daily food basket. One key question that needs to be addressed when building a model is how feasible it is to combine information from local grocery stores with the DGA to produce affordable and realistic diets. In addition, we must identify whether there are additional cost barriers that exist in specific communities which may hinder the ability to create affordable recommendations. A recommendation system must leverage information regarding local food availability and pricing in tandem with the DGA to produce healthy, affordable, and realistic daily diets.

In order to meet these challenges, we implement a case study using a linear programming optimization model that explores whether data can be leveraged to make affordable and healthy food purchase recommendations that meet federal dietary guidelines. In this study, we collect product-specific data from Kroger, a grocery store in the South Bend community, to produce an affordable and realistic daily food basket. We evaluate the realistic quality of our basket by determining whether the food products cover MyPlate requirements, have diverse categories, and contain independently consumable foods. In addition, we estimate price barriers for SNAP participants in affording a nutritious diet that meets federal dietary guidelines.

The goal of this paper is to demonstrate how the integration of data from local grocery stores and federal government databases can be used to assist specific communities in meeting their unique challenges. Our research is guided by these questions:

- Is it possible to integrate product nutritional information from local grocery stores with federal dietary guidelines to create a community-centered recommendation system?
- Is it possible to produce realistic recommendations within the SNAP allotment using this system?
- What barriers exist toward creating healthy and affordable recommendations?

Through our case study, we explore how to integrate information when focusing on community-centered optimizations. We also examine how additional constraints can be utilized in local grocery stores to influence the realistic quality of the daily diet recommendations. Our work incorporating cost analysis allows us to better understand the potential issues and complications regarding affordability of healthy diets when conducting future work. Our research contributes to a deeper understanding of the challenges involved with integrating local grocery store data into a food recommendation system. With our study, we analyze the effect of realism constraints on cost and demonstrate the feasibility of maintaining healthy diets within a constrained budget. This study bridges the gap between nutritious and affordable food choices and can serve as a valuable resource for community members, nutritionists, and those living in poverty or facing economic constraints in creating practical and budget friendly diet plans. The results from this community-centered case study could inform future work utilizing food information networks to aid in building more complex, larger-scale recommendation systems (Rong et al., 2006; Freyne and Berkovsky, 2010; Teng et al., 2012; Marshall, 2017; Schäfer et al., 2017; Trattner and Elsweiler, 2017; Pai, 2018; Ruis, 2019; Ceniza et al., 2020; Tian et al., 2022).

## 2. Materials and methods

The objective of the case study is to determine the minimal cost possible to achieve a diet that conforms to the DGA (U.S. Department of Agriculture and U.S. Department of Health and Human Services, 2020). In this section we provide an overview of the different components of our case study.

We collect publicly available data from a local grocery store and join it with nutritional information provided by the USDA in the Food Data Central (FDC) database (U.S. Department of Agriculture, Agricultural Research Service, 2022). We rely upon the nutrition facts label as we optimize around the recommended value of each of the macronutrients, minerals, and vitamins. The nutrition facts label is provided on most products and is implemented by the FDA as a policy tool to provide nutrition information (U.S. Food and Drug Administration and others, 1994).

Following data collection, we apply a Linear Programming model to create optimal budgets subject to the DGA. We test the model under three sets of constraints measuring the cost of maintaining a healthy diet under varying acceptability assumptions. Finally, we assess our models under a set of heuristics designed to evaluate the realism of each food basket, with a specific focus on the cost of each basket compared to the maximum monthly SNAP budget.

### 2.1. Data collection and preprocessing

Due to the growing concern of food insecurity in its low socio-economic neighborhoods, we focus on the Kroger store located in South Bend, IN (Dits, 2017). Kroger's Application Programming Interface (API) (Kroger Developers, 2022) provides details on available products at all Kroger locations in the United States. Using the API, we search for products containing common ingredients found in online recipes (Tian et al., 2021). For these products, we download the Universal Product Code (UPC), price, and availability in South Bend, IN. However, the product nutrition label information is not available through the API. Instead, we scraped Kroger's website for the nutrition label information for these products (Kroger, 2022). All information included in this study is up-to-date as of October 6, 2022.

Of the 24,293 products originally pulled from Kroger's API, only 16,206 (67%) had nutritional labels available to scrape. As Table 1 illustrates, the availability of nutritional information varied by product category. Categories such as “Produce” and “Deli” exclusively include food items but nearly a third do not include nutrition labels. The percentage of nutrition labels scraped is significantly smaller than other food categories such as “Canned and Packaged.” This seems to indicate that produce items such as fresh fruits, vegetables, and raw meat products may be underrepresented in our dataset due to a lack of available nutrition labels. This bias could be due to the voluntary requirement for grocers to include nutrition labels for raw produce, fish, and delicatessen-type food (U.S. Department of Health and Human Services, 2013). In addition, the original API dataset contains many non-food products which did not have nutritional labels.

**Table 1:** Number of products in the data downloaded from Kroger's API vs. the number of nutrition labels scraped from Kroger's website in each of the categories provided by Kroger.

**Kroger categories**	**API**	**Scraped**	**Percentage scraped**
Canned and packaged	1,647	1,513	91.9%
Breakfast	1,532	1,380	90.1%
Dairy	1,418	1,267	89.4%
Condiment and sauces	1,469	1,291	87.9%
Candy	778	678	87.1%
Frozen	2,411	2,054	85.2%
Meat and seafood	1,197	1,003	83.8%
International	795	662	83.3%
Pasta, sauces, grain	674	537	79.7%
Natural and organic	3,846	2,992	77.8%
Bakery	712	540	75.8%
Baby	389	291	74.8%
Beverages	2,773	2,064	74.4%
Snacks	2,385	1,733	72.7%
Baking goods	1,722	1,229	71.4%
Deli	562	386	68.7%
Produce	963	659	68.4%
Adult beverage	3	2	66.7%
Health	887	258	29.1%
Kitchen	143	8	5.6%
Apparel	81	4	4.9%
Garden and patio	112	3	2.7%
Cleaning products	786	14	1.8%
Beauty	740	10	1.4%
Personal care	1,044	11	1.1%
Pet care	767	8	1.0%
Home decor	154	0	0.0%
Entertainment	145	0	0.0%
Hardware	63	0	0.0%
Floral	6	0	0.0%
Sporting goods	10	0	0.0%
Party	7	0	0.0%
Halloween	9	0	0.0%
Automotive	4	0	0.0%
Electronics	1	0	0.0%
Office, school, and crafts	106	0	0.0%
Tobacco	3	0	0.0%

The percentage scraped column corresponds to the percentage of products in the original API dataset with a nutrition label available on Kroger. These categories are not mutually exclusive.

### 2.2. Data challenges

There are several challenges with the data obtained from Kroger that had to be addressed before it could be integrated with federal guidelines in a recommendation system. First, the nutritional information from Kroger contained inconsistent units of measurement. For example, the product “Kroger^Ⓡ^ 2% Reduced Fat Milk” has 5 mcg of Vitamin D but the DGA standard unit is in International Units (IU). Table 2 shows the percentage of products for each nutrient feature that are not listed in the DGA standard unit. As the Table illustrates, 20 of the 22 features required unit conversion to allow direct comparison of Kroger's nutrients to the DGA's nutrients. To address this, we converted all nutrient values to the unit listed in the DGA. This allows the Kroger products to be easily compared against the DGA.

**Table 2:** Percentage of products for each nutrient that are not listed in the DGA standard unit and the number of unique units present for each nutrient.

**Nutrient**	**Percentage**	**Number units**
Protein	0.04%	4
Total carbohydrate	0.09%	6
Dietary fiber	0.04%	2
Sugar	90.79%	2
Total fat	98.53%	6
Saturated fat	81.48%	3
Calcium	0.19%	4
Iron	0.17%	4
Magnesium	0.01%	2
Phosphorus	0%	1
Potassium	0.09%	3
Sodium	0.28%	4
Zinc	0.01%	2
Vitamin A	12.61%	6
Vitamin E	0.85%	4
Vitamin D	60.38%	6
Vitamin C	0.12%	4
Thiamin	0.01%	2
Riboflavin	0.01%	2
Niacin	0.02%	2
Vitamin K	0%	1
Folic acid	0.01%	2

Additionally, we performed data cleaning to achieve consistency in the number of servings within a product. The nutrition label includes the nutrition contents of a food product within a single serving. In order to compare the cost efficiency of two different products, it is necessary to know the number of servings within a product. Although the number of servings in a packaged item is occasionally included within the nutrition facts label, this information was missing in nearly 77% of the products within the Kroger dataset. We attempted to calculate the number of servings for each remaining product manually using the serving size included on the nutritional label as well as the size listed in the Kroger API. In ~18% of the products, there was insufficient information to calculate the number of servings so the number of servings was set to 1. While this assumption created some impractical results, the number of servings is only relevant within our case study as the denominator in the price per serving. Since our objective is to minimize cost per serving, an underestimate of the number of servings will result in an overestimate of the total cost which we deemed preferable to the alternative.

Finally, when checking the data scraped from Kroger we identified instances in which the nutritional label listed on the website directly contradicted an image of the label on the actual food product provided on the same webpage. For example, when searching for Kroger Blueberry Sausage & Pancake on a Stick, the user is presented with two conflicting nutritional labels. Despite the serving size being the same on both labels, the values for calories, total fat, sodium, and total carbohydrates do not match while other nutrients such as Saturated Fat and Added Sugars are missing entirely. Additionally, the unit of sodium listed on the product nutrition label in mg does not match the unit listed on the online nutrition label in g. Because of the problems with the nutritional information scraped from Kroger, we instead joined the Kroger information with nutrition information for branded products from the government database FDC with a one-to-one matching using the UPC.

Table 3 compares the alignment of FDC nutritional information against Kroger after converting all paired nutrients to the same unit. Overall, we generally see consistency between the two datasets, but there are still many cases in which the nutritional label from Kroger offers significantly different numbers than those from FDC as evidenced by the 50% column suggesting some large discrepancies between the two datasets. This implies that there are many cases where Kroger's information is not consistent with the USDA's.

**Table 3:** The percentage of products where the nutritional information from Kroger is within a percentage range of the nutritional information from FDC.

**Nutrient**	**0.0%**	**0.1%**	**1.0%**	**5.0%**	**10.0%**	**25.0%**	**50.0%**
Calories	19.6%	46.3%	71.5%	79.4%	84.1%	90.2%	93.5%
Protein	35.1%	73.5%	83.9%	84.7%	86.4%	90.4%	93.8%
Total lipid	39.2%	74.6%	82.4%	83.6%	85.5%	91.0%	94.1%
Carbohydrate	22.0%	71.6%	75.9%	80.5%	84.9%	89.6%	93.2%
Dietary fiber	48.0%	49.5%	66.0%	84.9%	86.1%	87.6%	90.6%
Calcium	41.0%	44.9%	61.4%	70.2%	72.4%	77.1%	83.9%
Iron	42.7%	52.3%	66.4%	71.7%	74.3%	79.3%	84.9%
Magnesium	95.7%	95.8%	96.2%	97.0%	97.6%	97.8%	98.1%
Phosphorus	95.6%	96.0%	96.3%	96.6%	96.7%	97.3%	98.2%
Potassium	39.7%	49.5%	64.4%	67.6%	69.7%	72.9%	76.6%
Sodium	21.7%	50.2%	65.2%	75.6%	81.3%	87.5%	91.8%
Zinc	96.7%	97.0%	97.2%	97.3%	97.4%	97.7%	98.5%
Vitamin A	81.9%	83.4%	84.5%	84.6%	84.8%	85.1%	86.7%
Vitamin D	90.4%	90.5%	90.9%	91.0%	91.0%	91.1%	91.2%
Vitamin E	97.0%	97.0%	97.1%	97.3%	97.3%	97.5%	97.7%
Vitamin C	84.2%	85.3%	87.3%	88.2%	88.4%	88.8%	90.4%
Thiamin	93.8%	93.8%	93.8%	93.9%	94.1%	94.9%	96.0%
Riboflavin	93.2%	94.1%	94.9%	95.2%	95.3%	96.9%	97.3%
Niacin	91.6%	93.6%	93.7%	93.8%	93.9%	96.5%	96.9%
Vitamin K	99.2%	99.3%	99.3%	99.3%	99.3%	99.4%	99.4%
Folic acid	96.7%	96.8%	96.9%	96.9%	96.9%	96.9%	96.9%
Added sugars	26.6%	30.5%	36.3%	38.5%	40.9%	46.2%	50.1%
Trans fat	96.1%	96.2%	96.9%	97.2%	97.2%	97.2%	97.3%
Saturated fat	49.4%	72.8%	84.4%	85.2%	85.8%	89.6%	92.6%

For example the 5.0% column represents the number of products where the Kroger value of each nutrient is within 5.0% of the FDC value for that nutrient.

**Figure 1:**
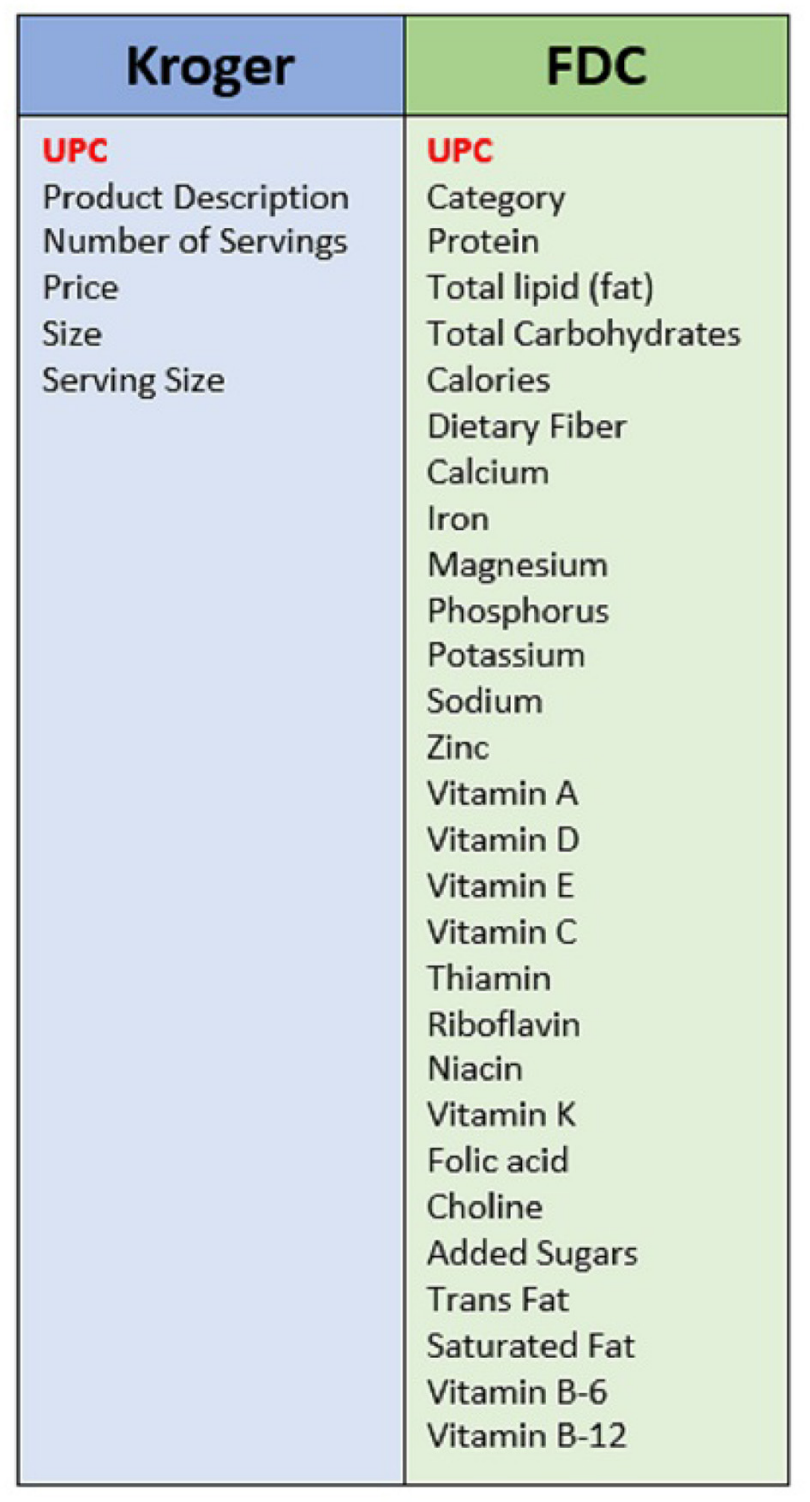
A list of all features within our dataset divided by the source used to acquire them.

After matching the information from FDC with that scraped from Kroger, we had a dataset of 10,777 products, 67% of the size of data scraped from Kroger. The final combined dataset consisted of food products available from the local Kroger store including the price and number of servings listed on Kroger's website and nutritional information including the amount of each nutrient from FDC. Figure 1 lists each of the features and their sources for our final dataset.

### 2.3. Linear programming formulation

Linear programming is a common optimization technique used to find a minimum or maximum value of a linear function subject to a number of constraints (Dantzig, 2002). Prior work has established using linear programming models for diet and recipe optimization (Briend et al., 2003; Donati et al., 2016; Elsweiler et al., 2017; Van Dooren, 2018; Brink et al., 2019). A linear programming model is particularly appropriate because it allows for easy integration of user preferences in the form of additional constraints which we refer to as “acceptability constraints.” Additionally, the flexibility of a linear programming model permits isolating individual constraints and performing a deep analysis into their impact on the recommendation. Observing the changes in the model output provides insight into how individual constraints may affect the final recommendation. Alternatively, by relaxing all constraints we obtain a more comprehensive understanding of how the various parameters interact with each other and are able to identify which nutrients are more restricting than others.

The case study is designed to examine whether SNAP participants are able to afford a healthy daily diet without exceeding their limited food budget. We created an integer programming model with the DGA's recommended intake of the vitamins and nutrients as constraints and the cost per serving of the products as the target value to be minimized. In other words, the linear programming model minimizes the cost of the diet while satisfying the DGA nutritional constraints. Mathematically, this model can be represented as:


(1) $$\begin{array}{l} \;\min\sum_{i=1}^{n}x_{i}*c_{i},x_{i}\in {\mathbb{Z}}_{\geq 0}\\ \text{subject to }v_{j_{min}}\leq \sum_{i=1}^{n}x_{i}*v_{j_{i}}\leq v_{j_{max}} \end{array}$$

Here, *x*_*i*_ is an integer variable corresponding to how many servings of a particular product, *i*, should be included in the optimal solution, *c*_*i*_ is the cost per serving of product *i* at the South Bend Kroger store, *v*_*j*_ is a specific vitamin or nutrient within the dietary guidelines, *v*_*j*_*min*__ and *v*_*j*_*max*__ are the respective minimum and maximum allowable intakes within the dietary guidelines, and *v*_*j*_*i*__ is the amount of nutrient *j* in product *i* according to the FDC.

The model selects a set of products where eating the specified number of servings per day will result in a diet conforming to the DGA while minimizing the financial burden on the consumer. The selected products within the dataset are represented by a non-negative integer variable representing the number of servings in the optimal solution where the unselected products are set to 0. By doing this, the unselected products will not contribute to the overall cost or nutrient values of the diet.

Because the nutritional information is provided per serving, optimizing around the total cost of a product would penalize larger “bulk” products which have more servings and may therefore cost more despite potentially being more nutritious than a similar, smaller product. To accommodate for this, all item prices were divided by the estimated number of servings previously calculated. The price per serving was used for the optimization value in the model.

This model was implemented utilizing the Mixed Integer Programming (MIP) python package (Toffolo and Santos, 2019). It is a variation of the Knapsack Problem and is NP-Complete. The MIP solver takes advantage of existing branch-and-cut methods to efficiently solve the integer programming problem. Furthermore, the solver is able to identify whether or not a solution is optimal or a best estimate. All results discussed within this paper were proven to be optimal by the solver.

#### 2.3.1. Establishing optimal daily diets

With the use of linear programming models, our work integrates data from the local grocery store with the DGA to attempt to produce optimal daily diets. The diets need to be both realistic and affordable for the user to accept and integrate the recommendations into their daily meal plans.

The first goal in our case study is to demonstrate that it is possible to produce an optimal diet under the DGA. The guidelines provide a range of each nutrient that a person should consume per day as part of a healthy diet. The specific ranges used as constraints in our optimization formula are shown in Table 4.

**Table 4:** Constraints used in linear programming model to enforce DGA in all three models.

	**Female 31–50**		**Male 31–50**	
**Nutrient**	**Min**	**Max**	**Min**	**Max**
Calories	1,600.0	2,200.0	2,200.0	3,000.0
Protein (kcal)	180.0	630.0	220.0	770.0
Protein (g)	46.0	N/A	56.0	N/A
Carbohydrates (kcal)	810.0	1,170.0	990.0	1,430.0
Carbohydrates (g)	130.0	N/A	130.0	N/A
Fiber (g)	25.0	N/A	31.0	N/A
Total lipid (kcal)	360.0	630.0	440.0	770.0
Calcium (mg)	1,000.0	N/A	1,000.0	N/A
Iron (mg)	18.0	N/A	8.0	N/A
Magnesium (mg)	320.0	N/A	420.0	N/A
Phosphorus (mg)	700.0	N/A	700.0	N/A
Potassium (mg)	2,600.0	N/A	3,400.0	N/A
Zinc (mg)	8.0	N/A	11.0	N/A
Vitamin A (mcg)	700.0	N/A	900.0	N/A
Vitamin E (mg)	15.0	N/A	15.0	N/A
Vitamin D (International Units)	600.0	N/A	600.0	N/A
Vitamin C (mg)	75.0	N/A	90.0	N/A
Thiamin (mg)	1.1	N/A	1.2	N/A
Riboflavin (mg)	1.1	N/A	1.3	N/A
Niacin (mg)	14.0	N/A	16.0	N/A
Vitamin K (mcg)	90.0	N/A	120.0	N/A
Folic acid (mcg)	400.0	N/A	400.0	N/A
Vitamin B-6 (mg)	1.3	N/A	1.3	N/A
Vitamin B-12 (mcg)	2.4	N/A	2.4	N/A
Added sugars (kcal)	N/A	180.0	N/A	220.0
Saturated fat (kcal)	N/A	180.0	N/A	220.0
Sodium (mg)	N/A	2,300.0	N/A	2,300.0

We developed 3 different models:

1. Model 1 searches the entire dataset of products for the minimal cost diet that satisfies the constraints from the DGA.
2. Model 2 uses the same DGA constraints but limits the dataset to food categories which are generally consumable on their own without having to be combined with another product. For example, the FDC category “baking additives and extracts” which contains products such as “Kroger Pure Baking Soda” is excluded in this model. The full list of categories that were included in Models 1 and 2 are provided in the Supplementary material Table S1 and Table S2.
3. Model 3 uses the same subset of the data as Model 2. However, in this model we also introduce new acceptability constraints along with the original DGA constraints. These constraints were designed to emulate MyPlate which promotes a diverse plate of protein foods, vegetables, fruits, dairy, and grain where protein foods includes both meat products and vegetarian protein foods such as legumes (U.S. Department of Agriculture, 2022). Additionally, they limited the maximum amount of servings of any other category to 2. The specific constraints added can be seen in Table 5. Due to the similarity of FDC categories, we manually condensed similar categories into a single overarching category. These condensed categories include the five MyPlate food groups to better represent MyPlate guidelines. For example, the categories “frozen vegetables,” “canned vegetables,” “vegetable based products/meals,” “pre-packaged fruit and vegetables,” and “vegetables prepared/processed” were combined into a single “vegetables” category. These new categories are listed in the Supplementary material Table S3 and Table S4.

**Table 5:** Acceptability constraints added in model 3.

**Category**	**Constraint type**	**Constraint**
Protein foods	Min	142 g (female); 170 g (male)
Vegetables	Min	2 servings (female); 3 servings (male)
Fruits	Min	2 servings (female); 3 servings (male)
Soup	Max	2 servings
Breakfast	Max	2 servings
Other frozen desserts	Max	2 servings
Snacks	Max	2 servings
Grains	Max	2 servings
Side dish	Max	2 servings
Dough based products/meals	Max	2 servings
Protein foods	Max	2 servings
Cooked and prepared	Max	2 servings
Frozen dinners and entrees	Max	2 servings
Other deli	Max	2 servings
Candy	Max	2 servings
Mexican dinner mixes	Max	2 servings
Chocolate	Max	2 servings
Pizza	Max	2 servings
Frozen bread and dough	Max	2 servings
Entrees, sides and small meals	Max	2 servings
Pizza mixes and other dry dinners	Max	2 servings
Prepared wraps and burittos	Max	2 servings
Prepared/preserved foods variety packs	Max	2 servings
Deli salads	Max	2 servings
Prepared subs and sandwiches	Max	2 servings

#### 2.3.2. Cost analysis

The second goal of the case study is to determine if any nutrients are more cost prohibitive than others. According to the Dietary Guidelines for Americans, one of the key elements toward eating healthier is reducing intake of sodium, saturated fat, and added sugars (U.S. Department of Agriculture and U.S. Department of Health and Human Services, 2020). We refer to these as the three areas of dietary concern. Each of these nutrients are linked to adverse health effects and the average American currently consumes significantly more than the daily recommendation (DiNicolantonio et al., 2016; Grillo et al., 2019). To better understand the ability of our model to reduce the overall cost of American's diets while increasing their healthy eating habits, we sought to establish a connection between cost and each of these individual nutrients. In other words, we set out to determine how the adjustment of the total allowance of these nutrients would affect the total diet cost.

Using the acceptability constraints in tandem with the DGA constraints as a starting point, we independently adjusted the maximum amount of each nutrient allowed in the model while holding everything else constant and observed how the overall price of the diet changed. For example, the recommended maximum daily intake of sodium for both males and females is 2,300 mg. We varied this maximum from a range of 1,000 to 5,000 mg in steps of 100. We repeated this for added sugar ranging from 50 to 400 kcals in steps of 10 and saturated fat from 0 to 350 kcals in steps of 10.

After determining how each of these three nutrients affected the cost independently, we examined how the interaction of the constraints could affect the overall price. If two nutrients were highly correlated with each other, altering their constraints independently could hide their impact on the cost of a diet. To address this, we simultaneously relaxed all constraints in the linear programming model by a given tolerance level. That is, if the tolerance level was 5% we lowered all minimum constraints by 5% from the DGA standard and increased all maximum constraints by 5%. We did this for a range of tolerance levels from 0% to 50%. After observing the optimization model at each tolerance level, we calculated the total amount of each nutrient in the optimization results and compared it to the original constraint established in the DGA.

### 2.4. Evaluation

To evaluate the affordability of a diet, we compare the cost of the recommended daily diet to the SNAP budget allotment for a single person household. The monthly SNAP benefits for a single person household is $250, or ~1$8.33 per day (Food and Nutrition Service U.S. Department of Agriculture, 2021). As our focus is on the SNAP participants within the local community, our goal is to produce diet recommendations that are under the daily SNAP allotment.

To evaluate the quality of a diet, we attempted to measure realism by following a set of heuristics we designed using MyPlate (U.S. Department of Agriculture, 2022) as a guide. The purpose of evaluating the realism of our daily diet is to determine whether the output reflects an “acceptable” daily meal plan. The evaluation will also help us to identify the effectiveness of different acceptability constraints.

1. Satisfaction of MyPlate food groups: MyPlate publishes an image of the recommended division of a plate into fruits, vegetables, grains, protein, and dairy. Additionally, the MyPlate guidelines include a number of cups of each of these food group categories that should be consumed per day. In each diet, we determine how many of these categories are contained within the results.
2. Overall diet diversity: In addition to the guidelines indicating the minimum amount necessary of each category, MyPlate also encourages variety in the diet. Specifically, the guidelines suggest varying vegetables and protein and ensuring that at least 50% of the fruits are whole fruits and that 50% of grains are whole grains (U.S. Department of Agriculture, 2022). Given this emphasis on diversity within the guidelines, we are looking for diets with a diverse set of food items for the consumer. We measure this by the number of unique products and categories contained in the diet.
3. Independently consumable foods: There are many food products within our dataset that may be useful cooking products but are not usually consumable as a standalone item. Because we are looking at a daily meal plan and not considering recipes, our goal is to suggest food items that are explicitly proteins, grains, fruits, or vegetables. Therefore, we consider the inclusion of these cooking products to be impractical for the purpose of this study.

To evaluate the cost impact of specific nutrients we observe the cost of each diet as the constraints for the three areas of concern are gradually relaxed. We compare the cost of the diet at the DGA threshold to the minimum cost diet. In addition, we evaluate at what constraint value the diet reaches the minimum cost.

Finally, to evaluate the effect of relaxing all constraints simultaneously, we determine which nutrients stay within the DGA thresholds despite the relaxed constraints. A nutrient falling outside the original constraint indicates that it is more difficult to affordably achieve a healthy intake level of the nutrient and therefore it will be more difficult to optimize for this in a recommendation system.

## 3. Results

The diets investigated in our case study are based on the DGA's daily nutritional goals for adult males and females in the 31–50 age group. According to the SNAP Quality Control Database, 21% of SNAP participants fall in the 31–50 age group which is the highest proportion of participants compared to other age ranges in the DGA (U.S. Department of Agriculture, Food and Nutrition Service, 2020).

### 3.1. Optimal daily diet results

Table 6 shows the optimal daily food basket produced using Model 1. These baskets show minimal food diversity. For example, the basket for females has three types of egg products (Eggland's best large white eggs, Eggland's best cage free large brown organic eggs, and Eggland's best extra large white eggs) while the basket for Males contains four servings of Carnation Breakfast Essentials Rich Milk Chocolate Nutritional Drink Mix. Overall, the female basket contains nine unique products and six unique categories while the male basket contains 11 unique products and nine unique categories. Neither basket contains any fruits. Additionally, categories such as “vegetable and cooking oils” and “milk additives” are shown in the output. While these products may be useful within a set of recipes, they fail to provide sustenance as standalone items in a daily diet. When we take into account all Kroger products with federal dietary constraints, we see that the output is unrealistic by our heuristics. The output produces multiple drinks and oil products leaving the only actual food as pasta, eggs, and beans which is not a substantial diet.

**Table 6:** Model 1: daily diet results using full dataset with DGA constraints.

**Number servings**	**Product**	**Category**	**Price per serving**
**(a) Females 31–50**			
2	Sun Vista^Ⓡ^ pinto beans	Canned and bottled beans	$0.03
3	Carnation breakfast essentials rich milk chocolate nutritional drink mix	Breakfast drinks	$0.55
1	Mazola corn oil	Vegetable and cooking oils	$0.07
1	Eggland's best large white eggs	Eggs and egg substitutes	$0.29
1	Eggland's best cage free large brown organic eggs	Eggs and egg substitutes	$0.50
2	Eggland's best extra large white eggs	Eggs and egg substitutes	$0.30
2	Kroger^Ⓡ^ spaghetti noodles	Pasta by shape and type	$0.12
2	Kroger^Ⓡ^ vitamin D whole milk	Milk	$0.20
1	Barilla^Ⓡ^ whole grain linguine pasta	Pasta by shape and type	$0.24
**Total price**			**$4.05**
**(b) Males 31–50**			
1	Bigelow^Ⓡ^ blackberry citrus herbal tea caffeine free tea bags plus zinc	Tea bags	$0.19
3	Sun Vista^Ⓡ^ pinto beans	Canned and bottled beans	$0.03
4	Carnation breakfast essentials rich milk chocolate nutritional drink mix	Breakfast drinks	$0.55
1	Kroger^Ⓡ^ sugar free hazelnut coffee creamer	Milk additives	$0.05
2	Mazola corn oil	Vegetable and cooking oils	$0.07
1	Eggland's best large white eggs	Eggs and egg substitutes	$0.29
2	Eggland's best extra large white eggs	Eggs and egg substitutes	$0.30
2	Kroger^Ⓡ^ spaghetti noodles	Pasta by shape and type	$0.12
1	Lipton raspberry iced tea mix	Powdered drinks	$0.01
2	Kroger^Ⓡ^ vitamin D whole milk	Milk	$0.20
1	Barilla^Ⓡ^ Whole grain linguine pasta	Pasta by shape and type	$0.24
**Total price**			**$4.45**

Table 7 shows the optimization results from Model 2. The female results select 18 servings of food from only nine product categories while the male results select 19 servings from only seven categories. Neither of the results contain any fruits and there is only a single vegetable in both results. Additionally the only dairy product is a single cheese slice for females and none for males. Considering the MyPlate food groups, we can see that at least three of the five categories are inadequately represented in this output. Also, while the outputs contain more unique products than the original results, the total number of categories represented is still relatively small. While this diet is more diverse than the one produced by Model 1, it still fails to meet our standards of a realistic result.

**Table 7:** Model 2: Daily diet results using filtered dataset with DGA constraints.

**Number servings**	**Product**	**Category**	**Price per serving**
**(a) Females 31–50**			
1	Raisin bran whole grain wheat and bran cereal	Cereal	$0.36
1	Kraft singles American cheese slices	Cheese	$0.21
1	Good to dough^Ⓡ^ enriched white bread	Breads and buns	$0.05
1	Planters^Ⓡ^ dry roasted peanuts	Popcorn, peanuts, seeds, and related snacks	$0.23
1	Eggland's best cage free large brown organic eggs	Eggs and egg substitutes	$0.50
3	Eggland's best extra large white eggs	Eggs and egg substitutes	$0.30
3	Kroger^Ⓡ^ spaghetti noodles	Pasta by shape and type	$0.12
1	Dole spinach	Pre-packaged fruit and vegetables	$0.86
3	Welch's value size mixed fruit snacks	Candy	$0.22
1	Tasty chicken tikka masala dinner kit	Cereal	$0.60
1	Kroger^Ⓡ^ salted with sea salt peanuts	Popcorn, peanuts, seeds, and related snacks	$0.17
1	Kroger^Ⓡ^ Spanish salted with sea salt peanuts	Popcorn, peanuts, seeds, and related snacks	$0.17
**Total price**			**$5.07**
**(b) Males 31–50**			
2	Raisin bran whole grain wheat and bran cereal	Cereal	$0.36
1	Good to Dough^Ⓡ^ enriched white bread	Breads and buns	$0.05
1	Zero king size candy bar	Candy	$0.01
1	Planters^Ⓡ^ dry roasted peanuts	Popcorn, peanuts, seeds, and related snacks	$0.23
1	Eggland's best cage free large brown organic eggs	Eggs and egg substitutes	$0.50
3	Eggland's best extra large white eggs	Eggs and egg substitutes	$0.30
3	Kroger^Ⓡ^ spaghetti noodles	Pasta by shape and type	$0.12
1	Dole spinach	Pre-packaged fruit and vegetables	$0.86
3	Welch's value size mixed fruit snacks	Candy	$0.22
1	Tasty chicken tikka masala dinner kit	Cereal	$0.60
2	Kroger^Ⓡ^ salted with sea salt peanuts	Popcorn, peanuts, seeds, and related snacks	$0.17
**Total price**			**$5.23**

Table 8 contains the results from Model 3. In both cases, the optimal result contains 15 unique products, an increase from all previous outputs. Overall, 12 categories are represented in the Female diet while 14 are represented in the Male diet providing a more diverse output. In addition, this basket has more products from vegetables, fruits, and dairy which were largely absent in the previous results while maintaining a selection of grains and protein. We consider these baskets to be more realistic than those from the previous models.

**Table 8:** Model 3: Daily diet results using filtered dataset with DGA constraints and acceptability constraints.

**Number servings**	**Product**	**Category**	**Price per serving**
**(a) Females 31–50**			
1	Blue Diamond^Ⓡ^ honey roasted almonds	Popcorn, peanuts, seeds, and related snacks	$0.62
1	Blue Diamond^Ⓡ^ lightly salted almonds	Popcorn, peanuts, seeds, and related snacks	$1.29
1	Kellogg's special K original breakfast cereal family size	Cereal	$0.61
1	Kroger^Ⓡ^ pineapple chunks in pineapple juice	Canned fruit	$0.28
1	Kroger^Ⓡ^ whole berry cranberry sauce	Canned fruit	$0.26
1	Kroger^Ⓡ^ no salt added cut green beans	Canned vegetables	$0.25
1	Sun Vista^Ⓡ^ Pinto beans	Canned and bottled beans	$0.03
2	Carnation breakfast essentials rich milk chocolate drink	Candy	$1.33
1	Hunt's four cheese pasta sauce	Prepared pasta and pizza sauces	$0.22
1	Eggland's best extra large white eggs	Eggs and egg substitutes	$0.30
1	Kroger^Ⓡ^ spaghetti noodles	Pasta by shape and type	$0.12
1	Dole spinach	Pre-packaged fruit and vegetables	$0.86
1	Honey nut cheerios gluten free breakfast cereal	Cereal	$0.29
1	Roth^Ⓡ^ original havarti cheese	Cheese	$0.05
2	Flavor ice giant freezer pops	Ice cream and frozen yogurt	$0.09
**Total price**			**$8.02**
**(b) Males 31–50**			
1	Blue Diamond^Ⓡ^ honey roasted almonds	Popcorn, peanuts, seeds, and related snacks	$0.62
1	Blue Diamond^Ⓡ^ lightly salted almonds	Popcorn, peanuts, seeds, and related snacks	$1.29
1	Kellogg's special K original breakfast cereal family size	Cereal	$0.61
3	Kroger^Ⓡ^ whole berry cranberry sauce	Canned fruit	$0.26
1	Sun Vista^Ⓡ^ pinto beans	Canned and bottled beans	$0.03
2	Carnation breakfast essentials rich milk chocolate drink	Candy	$1.33
1	Ragu chunky tomato garlic and Onion pasta sauce	Prepared pasta and pizza sauces	$0.35
1	La Banderita white corn tortillas	Mexican dinner mixes	$0.12
1	Eggland's best extra large white eggs	Eggs and egg substitutes	$0.30
1	Kroger^Ⓡ^ spaghetti noodles	Pasta by shape and type	$0.12
1	Dole spinach	Pre-packaged fruit and vegetables	$0.86
2	Muir Glen organic tomato puree	Vegetables prepared/processed	$0.27
1	Honey nut cheerios gluten free breakfast cereal	Cereal	$0.29
1	Roth^Ⓡ^ original havarti cheese	Cheese	$0.05
2	Flavor ice giant freezer pops	Ice cream and frozen yogurt	$0.09
**Total price**			**$8.80**

### 3.2. Cost analysis results

**Figure 2:**
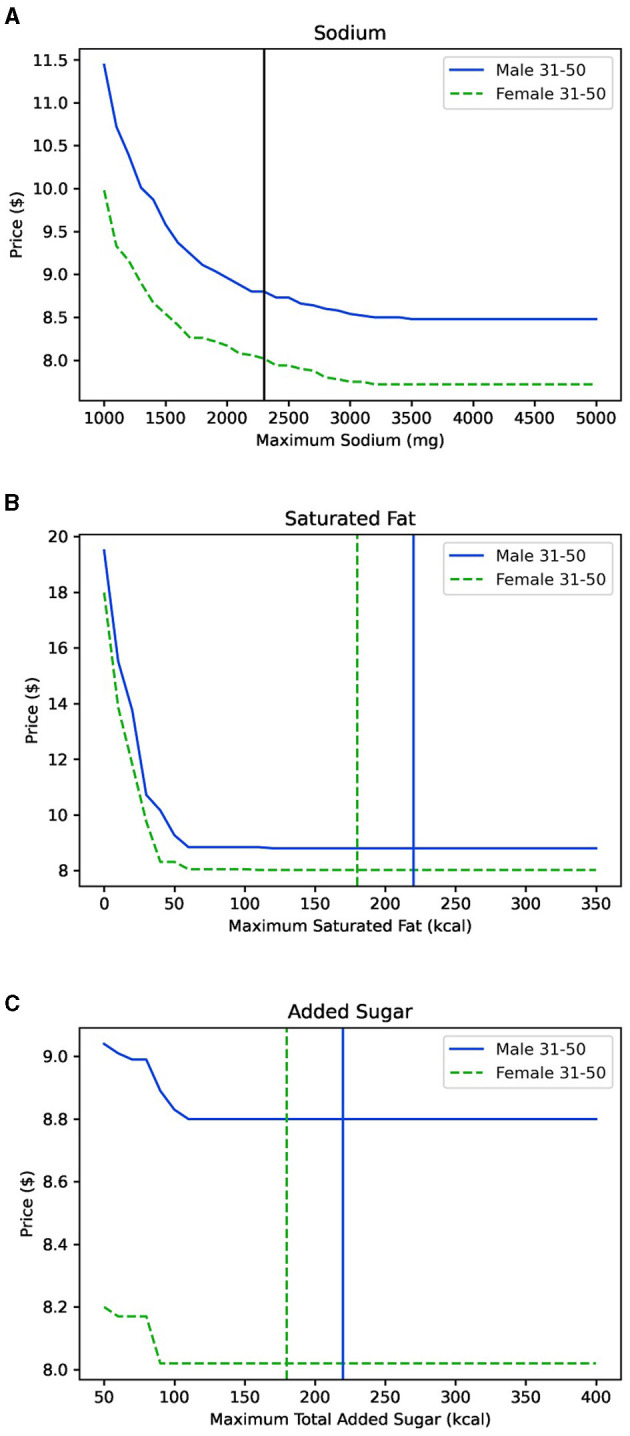
In each of these graphs, the *x*-axis corresponds to the maximum amount of the specified nutrient allowed within the constraints and the *y*-axis is the price of the optimal basket generated. The verical lines represent the DGA threshold for males (blue), females (green), or (black). **(A)** Relaxing the tolerance for sodium within the DGA can result a slightly lower cost basket. **(B)** Relaxing the tolerance for saturated fat does not lower the price of the optimal healthy basket for males or females. **(C)** Relaxing the tolerance for added sugar does not lower the price of the optimal healthy basket for males or females.

Having established parameters within the optimization model, the next step is to explore the cost of various nutrients. The DGA identify three specific nutrients which Americans currently overconsume: sodium, saturated fat, and added sugars. We sought to answer the question: How does relaxing these constraints affect the overall cost of the basket? Specifically, for each nutrient we varied the maximum constraint over a range of values while holding all other constraints constant. Figure 2 shows the results. In each of these graphs, the average American is currently consuming more than the daily guideline (to the right of the vertical lines). The goal of a recommendation model would be to produce diets with constraints lower than the DGA guideline (to the left of the line) without significantly increasing the price.

In Figure 2A, the male recommended diet costs $8.80 at the daily recommended maximum before reaching its minimum value of $8.48 at 3,500 mg. The Female recommended diet costs $8.02 at the daily recommended maximum before reaching its minimum value of $7.72 at 3,200 mg. Sodium appears to have a direct impact on cost as the price of the optimal diet continues to decrease as it passes the vertical line in the maximum sodium allowance. While the line flattens out between 3,000 and 3,500 mg, which is notably still lower than the average American adult consumption, it does so at a cost 30–40 cents cheaper than the healthy diet, nearly 5% savings.

In Figures 2B, C, the cost flattens out at a level below the recommended maximum daily intake of saturated fat or added sugars. Notably, neither macronutrient appear to have any effect on price. The optimal cost basket can be achieved with less of each nutrient than the daily guidelines as represented by the line flattening out prior to the vertical line.

Finally, we examine the interaction of the nutrients by relaxing all constraints simultaneously. We compare the amount of each nutrient in the optimal diet against the standard guideline value as shown in Table 9. In these tables a row with False values represents a nutrient which is more difficult to optimize within the DGA.

**Table 9:** Constraint relaxation results.

	**0%**	**2.5%**	**5%**	**10%**	**15%**	**20%**	**25%**	**30%**	**40%**	**50%**
**(a) Females 31–50**										
Cost	$8.02	$7.94	$7.80	$6.99	$6.69	$6.02	$5.42	$5.11	$4.22	$3.53
Protein	TRUE	TRUE	TRUE	TRUE	TRUE	TRUE	TRUE	TRUE	TRUE	TRUE
Total carbohydrates	TRUE	TRUE	TRUE	TRUE	TRUE	TRUE	TRUE	TRUE	TRUE	TRUE
Fiber	TRUE	TRUE	TRUE	TRUE	TRUE	FALSE	TRUE	FALSE	FALSE	FALSE
Added sugars	TRUE	TRUE	TRUE	TRUE	TRUE	TRUE	TRUE	TRUE	TRUE	TRUE
Total lipid	TRUE	TRUE	TRUE	TRUE	TRUE	TRUE	TRUE	TRUE	TRUE	TRUE
Saturated fat	TRUE	TRUE	TRUE	TRUE	TRUE	FALSE	FALSE	FALSE	TRUE	FALSE
Calcium	TRUE	TRUE	TRUE	FALSE	TRUE	TRUE	TRUE	TRUE	FALSE	FALSE
Iron	TRUE	TRUE	TRUE	TRUE	TRUE	TRUE	TRUE	TRUE	TRUE	TRUE
Magnesium	TRUE	TRUE	TRUE	TRUE	TRUE	FALSE	FALSE	FALSE	FALSE	FALSE
Phosphorus	TRUE	TRUE	FALSE	FALSE	FALSE	FALSE	FALSE	FALSE	FALSE	FALSE
Potassium	TRUE	TRUE	TRUE	FALSE	FALSE	FALSE	TRUE	TRUE	FALSE	FALSE
Sodium	TRUE	TRUE	TRUE	FALSE	FALSE	FALSE	TRUE	TRUE	TRUE	TRUE
Zinc	TRUE	TRUE	TRUE	TRUE	FALSE	TRUE	FALSE	FALSE	FALSE	FALSE
Vitamin A	TRUE	TRUE	TRUE	TRUE	TRUE	TRUE	TRUE	TRUE	TRUE	TRUE
Vitamin E	TRUE	TRUE	TRUE	FALSE	TRUE	FALSE	FALSE	FALSE	FALSE	FALSE
Vitamin D	TRUE	FALSE	FALSE	FALSE	FALSE	FALSE	FALSE	FALSE	FALSE	FALSE
Vitamin C	TRUE	TRUE	TRUE	FALSE	TRUE	TRUE	FALSE	TRUE	FALSE	FALSE
Thiamin	TRUE	TRUE	TRUE	TRUE	TRUE	TRUE	TRUE	TRUE	TRUE	TRUE
Riboflavin	TRUE	TRUE	TRUE	TRUE	TRUE	TRUE	TRUE	TRUE	TRUE	TRUE
Niacin	TRUE	TRUE	TRUE	TRUE	TRUE	TRUE	TRUE	TRUE	FALSE	TRUE
Vitamin K	TRUE	TRUE	TRUE	TRUE	TRUE	TRUE	TRUE	TRUE	TRUE	TRUE
Folic acid	TRUE	TRUE	TRUE	FALSE	FALSE	FALSE	FALSE	FALSE	FALSE	FALSE
Vitamin B-6	TRUE	TRUE	TRUE	TRUE	TRUE	TRUE	TRUE	FALSE	FALSE	FALSE
Vitamin B-12	TRUE	TRUE	TRUE	TRUE	TRUE	TRUE	TRUE	TRUE	TRUE	TRUE
**(b) Males 31–50**										
Cost	$8.80	$8.73	$8.65	$8.20	$7.50	$7.23	$6.78	$6.49	$4.83	$4.19
Protein	TRUE	TRUE	TRUE	TRUE	TRUE	TRUE	TRUE	TRUE	TRUE	TRUE
Total carbohydrates	TRUE	TRUE	TRUE	TRUE	TRUE	TRUE	TRUE	TRUE	TRUE	TRUE
Fiber	TRUE	TRUE	TRUE	FALSE	FALSE	TRUE	TRUE	TRUE	TRUE	FALSE
Added sugars	TRUE	TRUE	TRUE	FALSE	FALSE	TRUE	FALSE	TRUE	TRUE	TRUE
Total lipid	TRUE	TRUE	TRUE	TRUE	TRUE	TRUE	TRUE	TRUE	TRUE	TRUE
Saturated fat	TRUE	TRUE	TRUE	FALSE	FALSE	TRUE	TRUE	FALSE	FALSE	FALSE
Calcium	TRUE	TRUE	TRUE	TRUE	TRUE	TRUE	TRUE	TRUE	TRUE	FALSE
Iron	TRUE	TRUE	TRUE	TRUE	TRUE	TRUE	TRUE	TRUE	TRUE	TRUE
Magnesium	TRUE	TRUE	FALSE	FALSE	FALSE	TRUE	FALSE	FALSE	FALSE	FALSE
Phosphorus	TRUE	TRUE	FALSE	FALSE	FALSE	TRUE	TRUE	FALSE	FALSE	FALSE
Potassium	TRUE	FALSE	FALSE	FALSE	FALSE	FALSE	FALSE	FALSE	FALSE	FALSE
Sodium	TRUE	FALSE	FALSE	FALSE	FALSE	FALSE	FALSE	FALSE	TRUE	FALSE
Zinc	TRUE	TRUE	TRUE	TRUE	TRUE	TRUE	FALSE	FALSE	FALSE	FALSE
Vitamin A	TRUE	TRUE	TRUE	TRUE	TRUE	TRUE	TRUE	TRUE	TRUE	TRUE
Vitamin E	TRUE	TRUE	TRUE	TRUE	TRUE	FALSE	FALSE	FALSE	FALSE	FALSE
Vitamin D	TRUE	FALSE	FALSE	FALSE	FALSE	FALSE	FALSE	FALSE	FALSE	FALSE
Vitamin C	TRUE	TRUE	TRUE	TRUE	TRUE	TRUE	TRUE	TRUE	FALSE	FALSE
Thiamin	TRUE	TRUE	TRUE	TRUE	TRUE	TRUE	FALSE	TRUE	TRUE	TRUE
Riboflavin	TRUE	TRUE	TRUE	TRUE	TRUE	TRUE	TRUE	TRUE	TRUE	TRUE
Niacin	TRUE	TRUE	TRUE	TRUE	TRUE	TRUE	TRUE	FALSE	TRUE	TRUE
Vitamin K	TRUE	TRUE	TRUE	TRUE	TRUE	TRUE	TRUE	TRUE	TRUE	TRUE
Folic acid	TRUE	TRUE	TRUE	FALSE	FALSE	FALSE	FALSE	FALSE	FALSE	FALSE
Vitamin B-6	TRUE	TRUE	TRUE	TRUE	TRUE	TRUE	TRUE	TRUE	TRUE	FALSE
Vitamin B-12	TRUE	TRUE	TRUE	TRUE	TRUE	TRUE	TRUE	TRUE	TRUE	TRUE

The *x*-axis shows the percentage by which all constraints were relaxed.

A value of TRUE identifies a nutrient whose value in the optimal basket falls within the original DGA constraints. A FALSE identifies a nutrient whose value falls outside the original DGA constraints indicating it is more difficult to optimize for this nutrient.

## 4. Discussion

Through our case study, we demonstrate the potential of building a community-centered recommendation system that integrates information from grocery stores with federal dietary guidelines. The case study demonstrates that we can use this information at a community level to assist consumers in finding affordable and healthy food options that meet their dietary needs. Creating diet recommendations with products available in their community gives them a personalized, actionable plan which fits the nutritional guidelines. Through our research we observe that it is possible to have healthy and realistic diets that are affordable for those who rely on SNAP benefits. In doing so, we highlight some of the practical challenges that exist in the creation of local-community recommendations, such as the issues with data integration, taxonimization of categories, and availability of products for dietary fulfillment.

The optimal daily diets produced in the results indicate that it is possible to achieve a daily diet within the monthly SNAP budget of $250 for a single household, or ~1$8.33 per day (Food and Nutrition Service U.S. Department of Agriculture, 2021). Our results show that Model 1 and Model 2 produced a diet for males and females that is under the SNAP daily budget. In Model 3, although the female diet is within the SNAP budget, the cost of the male diet is $8.80 which is slightly above the SNAP budget.

In addition, our results show that adding acceptability constraints to the model produces diets that are more realistic which contain a diverse set of product categories. Model 1, without any acceptability constraints, produces a diet with minimal food diversity and with items that fail to provide sustenance on their own. The lack of constraints also produces fewer unique products which is an observation consistent with previous studies (Conforti and D'Amicis, 2000). The addition of acceptability constraints produces a more realistic diet with more diverse product categories and with more products that are representative of MyPlate food groups.

While difficult to apply, the application of acceptability constraints has been shown by other literature to assist with creating realistic linear programming outcomes (Parlesak et al., 2016; Van Dooren, 2018; Toledo et al., 2019). However, as we have seen both within our case study and in other optimization studies, introducing acceptability constraints can have a significant impact on the price of the basket (Maillot et al., 2010; Donati et al., 2016; Parlesak et al., 2016). Specifically, in our case study we show that adding acceptability constraints produces a budget that is higher than the daily SNAP allotment. Future research will need to consider that the application of acceptability constraints may lead to more expensive diets and may not be feasible for SNAP participants to afford.

Furthermore, our study focuses primarily on analyzing affordable diets compared to SNAP budgets, but considering the influence of other food assistance programs, such as the Special Supplemental Nutrition Program for Women, Infants, and Children (WIC), could add depth to the interpretation of our model results. WIC's unique food package encourages healthier choices like fruits, vegetables, whole grains, and lean proteins. Dual recipients of SNAP and WIC could potentially have access to a wider range of nutritious products, which could moderate the impact of price point changes observed in our models. In future models, additional constraints for these WIC benefits could be introduced to ensure they fully leverage their benefits. Incorporating additional constraints for WIC benefits could allow users to maintain a healthy and diverse diet while fully expending the resources available to them. However, handling the WIC benefits could be a challenge due to the inconsistency in the measurement of the constraints. Some product benefits are in dollar amounts while others are in weights or quantities. This variation in the measurement presents a challenge given the limitations of our dataset and linear programming model. In the future, we would like to explore a more holistic perspective that integrates the dynamics of multiple food assistance programs that could enhance the realism and adaptability of these dietary models.

Our study also indicates that a future recommendation system will need to better integrate user preferences. One key issue in accomplishing this is the lack of a quality taxonimization of products. While both Kroger and FDC provide product categories, they are incomplete and difficult to employ. These categories are not at the level of granularity needed to accurately represent a user's preferences. One example is that there are multiple categories for poultry, but there are no categories for other specific meat products such as beef or pork. This creates limitations within the model for users who may have preferences for a specific type of meat. These categories are how we currently introduce acceptability and more granular information could allow for finer-tuned constraints that more accurately portray user preferences. Additionally, there does not currently exist an accepted method to analyze the realism of a diet short of user feedback. While the heuristics used in this paper were well suited for our purposes, better product categorization would allow one to more precisely analyze the overall quality of a diet within a user's preferences.

Our results explore potential cost barriers that may limit future optimization models' ability to find affordable recommendations. Through our case study, we demonstrate that an affordable diet can be attained without concern for added sugars or saturated fat. The results indicate that neither of these nutrients is considerably expensive and that it is possible to create recommendations within the DGA without sacrificing cost. On the other hand, the opposite is seen for sodium. Our results indicate that in order to create recommendations which satisfy the sodium DGA, consumers may have to consider a higher cost diet. This finding presents a unique challenge when creating future recommendation systems. Lower sodium foods tend to be less expensive and have a longer shelf life than alternatives but are less healthy. This also suggests that it is more challenging for individual users with strict budget restrictions to make changes to their eating habits which satisfy the DGA as many of these cheaper foods are processed foods which have sodium being added at the manufacturer level. In future recommendation systems, it will be important to consider this trade-off between health and budget and attempt to best optimize around a specific user's needs.

In addition to these three areas of concern, our results examine the interaction between nutrients to further identify whether any particular nutrient is relatively more expensive. The results indicate that many of the vitamin and mineral daily goals are difficult to achieve. For example, as seen in Table 9 Phosphorus and Vitamin D are difficult for both men and women to achieve. Notably, many vitamins and minerals are difficult to acquire because of their rarity in products. Table 10 shows the percentage of products within our final dataset where the value of the nutrient is equal to 0. As shown, many of the vitamins and minerals are absent in over 90% of the products while a more prominent macronutrient, such as sodium, is absent in only ~112% of products. This indicates that greater availability of these nutrients through better product offerings could assist Americans in achieving a healthy diet.

**Table 10:** Percentage of Products where the value of the nutrient is equal to 0.

**Nutrient**	**Percent zero**
Protein	26.66%
Total carbohydrates	10.28%
Fiber	44.56%
Added sugars	65.8%
Total lipid	30.79%
Saturated fat	44.29%
Calcium	41.27%
Iron	40.24%
Magnesium	96.17%
Phosphorus	96.03%
Potassium	47.75%
Sodium	12.09%
Zinc	97.17%
Vitamin A	87.26%
Vitamin E	99.15%
Vitamin D	97.68%
Vitamin C	85.25%
Thiamin	97.17%
Riboflavin	93.94%
Niacin	92.27%
Vitamin K	99.39%
Folic acid	96.81%
Vitamin B-6	95.23%
Vitamin B-12	96%

While our study offers valuable insights into diet optimization, there are some limitations to our method which could be addressed in future work. Linear Programming requires linear constraints which make it difficult to capture the complexities of dietary guidelines. For example, the constraints within our model treat macronutrients and micronutrients independently, when an approach that considers their balance within a diet may be more suitable. Furthermore, our results do not take into account meal preparation methods or the shelf life of food items which would be important considerations in practice. Finally, we utilized a daily diet model that operates under the assumption of a flat budget per day. With the current economic and food landscapes affecting grocery prices and product availability, a more dynamic approach which anticipates the user's needs over a longer time frame may be more suitable.

Our results demonstrate the benefits in building a community-centric recommendation system. We propose that through the integration of local grocery store data with federal guidelines, we are able to create diet recommendations that are both nutritious and affordable within the SNAP allocation. These diets provide a daily meal plan that could improve health outcomes for low-income communities. We believe that recommendation systems can further incorporate data from local communities with government databases to directly address their specific health and economic challenges. With a better understanding of the obstacles encountered in the building of community-focused models, our work will contribute toward the development of broader food information networks for future recommendation systems. Our work demonstrates possible solutions for those suffering food insecurity in other low-income communities that may be used as a stepping stone in creating a more global food information network for future recommendation systems.

## Data availability statement

Publicly available datasets were analyzed in this study. This data can be found at: https://fdc.nal.usda.gov/download-datasets.html, https://developer.kroger.com/reference/.

## Author contributions

JG and AS developed and designed the case study and managed the writing of this manuscript. HE-M, RM, and NC provided significant mentoring and feedback throughout the project. All authors reviewed and approved the manuscript.
