# Corrigendum: A community focused approach toward making healthy and affordable daily diet recommendations

**DOI:** 10.3389/fdata.2024.1396638

**Published:** 2024-04-04

**Authors:** Joe Germino, Annalisa Szymanski, Heather A. Eicher-Miller, Ronald Metoyer, Nitesh V. Chawla

**Affiliations:** ^1^Department of Computer Science and Engineering, Lucy Family Institute, University of Notre Dame, Notre Dame, IN, United States; ^2^Department of Nutrition Science, Purdue University, West Lafayette, IN, United States

**Keywords:** linear programming, food recommendation, diet cost, community-centered optimization, food information networks, nutrition

In the published article, there was an error in the author list, and author Heather A. Eicher-Miller was erroneously excluded. The corrected author list appears below.

Joe Germino^†^, Annalisa Szymanski^†^, Heather A. Eicher-Miller, Ronald Metoyer, and Nitesh V. Chawla^*^

Heather A. Eicher-Miller was not included as an author in the published article. The corrected Author Contributions Statement appears below.

JG and AS developed and designed the case study and managed the writing of this manuscript. HE-M, RM, and NC provided significant mentoring and feedback throughout the project. All authors reviewed and approved the manuscript.

The authors apologize for this error and state that this does not change the scientific conclusions of the article in any way. The original article has been updated.

