# Supplementary material for: A community focused approach toward making healthy and affordable daily diet recommendations
Source: Front Big Data. 2023 Nov 6;6:1086212. doi: 10.3389/fdata.2023.1086212 (PMC10661405; doi:10.3389/fdata.2023.1086212)
Supplement: Supplementary file 1 [file Table_1.pdf]

Supplementary Material

1 SUPPLEMENTARY TABLES AND FIGURES

Table S1. This table provides the FDC food categories that are used in Model 1 of the case study.

Cheese	Seasoning Mixes, Salts, Marinades & Tenderizers	Frozen Fish & Seafood
Fruit & Vegetable Juice, Nectars & Fruit Drinks	Alcohol	Other Frozen Meats
Milk Additives	Sauces/Spreads/Dips/Condiments	Herbs/Spices/Extracts
Prepared Soups	Frozen Dinners & Entrees	Meat/Poultry/Other Animals Sausages Prepared/Processed
Frozen Vegetables	Other Deli	Pasta/Noodles
Ice Cream & Frozen Yogurt	Flours & Corn Meal	Pepperoni, Salami & Cold Cuts
Canned Condensed Soup	Cake, Cookie & Cupcake Mixes	Canned Soup
Soda	Grains/Flour	Non Alcoholic Beverages Ready to Drink
Cereal	Nut & Seed Butters	Water
Other Frozen Desserts	Cookies & Biscuits	Chewing Gum & Mints
Baking Decorations & Dessert Toppings	Candy	Frozen Breakfast Sandwiches, Biscuits & Meals
Salad Dressing & Mayonnaise	Crackers & Biscotti	Breakfast Sandwiches, Biscuits & Meals
Snack, Energy & Granola Bars	Mexican Dinner Mixes	Sweet Bakery Products
Chips, Pretzels & Snacks	Bread & Muffin Mixes	Prepared Wraps and Burittos
Cakes, Cupcakes, Snack Cakes	Other Drinks	Flavored Rice Dishes
Yogurt	Chocolate	Liquid Water Enhancer
Dips & Salsa	Confectionery Products	Iced & Bottle Tea
Cream	Baking/Cooking Mixes/Supplies	Energy, Protein & Muscle Recovery Drinks
Baking Additives & Extracts	Pastry Shells & Fillings	Frozen Pancakes, Waffles, French Toast & Crepes
Prepared Pasta & Pizza Sauces	Wholesome Snacks	Sport Drinks
Frozen Appetizers & Hors D'oeuvres	Other Cooking Sauces	Frozen Sausages, Hotdogs & Brats
Other Snacks	Jam, Jelly & Fruit Spreads	Frozen Poultry, Chicken & Turkey
Lunch Snacks & Combinations	Frozen Fruit & Fruit Juice Concentrates	Bread
Canned Vegetables	Biscuits/Cookies	Frozen Bacon, Sausages & Ribs
Dough Based Products / Meals	Pizza	Meat/Poultry/Other Animals Prepared/Processed
Pasta Dinners	Fruit Prepared/Processed	Tea Bags
Frozen Patties and Burgers	Frozen Bread & Dough	Coffee/Tea/Substitutes
Flavored Snack Crackers	Vegetable Based Products / Meals	Savoury Bakery Products
Snacks	Gravy Mix	Granulated, Brown & Powdered Sugar
Popcorn, Peanuts, Seeds & Related Snacks	Vegetable & Cooking Oils	Pancakes, Waffles, French Toast & Crepes
Milk	Pre-Packaged Fruit & Vegetables	Canned Meat
Plant Based Milk	Pickles, Olives, Peppers & Relishes	All Noodles
Pasta by Shape & Type	Herbs & Spices	Grain Based Products / Meals
Other Meats	Bacon, Sausages & Ribs	Other Condiments
Poultry, Chicken & Turkey	Yogurt/Yogurt Substitutes	Stuffing
Meat/Poultry/Other Animals Unprepared/Unprocessed	Entrees, Sides & Small Meals	Fish & Seafood
Cooked & Prepared	Frozen Prepared Sides	Eggs & Egg Substitutes
Ketchup, Mustard, BBQ & Cheese Sauce	Vegetable and Lentil Mixes	Dairy/Egg Based Products / Meals
Oriental, Mexican & Ethnic Sauces	Crusts & Dough	Coffee
Syrups & Molasses	Pizza Mixes & Other Dry Dinners	Fish Unprepared/Unprocessed
Other Soups	Vegetarian Frozen Meats	Specialty Formula Supplements
Croissants, Sweet Rolls, Muffins & Other Pastries	Processed Cereal Products	French Fries, Potatoes & Onion Rings
Breads & Buns	Powdered Drinks	Butter & Spread
Rice	Canned Seafood	Other Grains & Seeds
Chili & Stew	Prepared/Preserved Foods Variety Packs	Honey
Tomatoes	Canned Tuna	Baby/Infant Foods/Beverages
Vegetables Prepared/Processed	Puddings & Custards	Deli Salads
Canned Fruit	Plant Based Water	Vitamins
Canned & Bottled Beans	Breakfast Drinks	Baking
Sausages, Hotdogs & Brats	Gelatin, Gels, Pectins & Desserts	Weight Control
Butter/Butter Substitutes	Sweet Spreads	Meal Replacement Supplements
Bread/Bakery Products Variety Packs	Nuts/Seeds - Prepared/Processed (Shelf Stable)	Desserts/Dessert Sauces/Toppings
Oils Edible	Cereals Products - Ready to Eat (Shelf Stable)	Health Care
Prepared Subs & Sandwiches		

Table S2. This table provides the FDC food categories that are used in Model 2 of the case study.

Cheese	Frozen Prepared Sides
Prepared Soups	Vegetable and Lentil Mixes
Frozen Vegetables	Pizza Mixes & Other Dry Dinners
Ice Cream & Frozen Yogurt	Other Soups
Canned Condensed Soup	Croissants, Sweet Rolls, Muffins & Other Pastries
Cereal	Breads & Buns
Other Frozen Desserts	Rice
Snack, Energy & Granola Bars	Chili & Stew
Chips, Pretzels & Snacks	Vegetables Prepared/Processed
Cakes, Cupcakes, Snack Cakes	Canned Fruit
Yogurt	Canned & Bottled Beans
Dips & Salsa	Sausages, Hotdogs & Brats
Prepared Pasta & Pizza Sauces	Frozen Fish & Seafood
Frozen Appetizers & Hors D'oeuvres	Other Frozen Meats
Other Snacks	Meat/Poultry/Other Animals Sausages Prepared/Processed
Lunch Snacks & Combinations	Pasta/Noodles
Canned Vegetables	Pepperoni, Salami & Cold Cuts
Dough Based Products / Meals	Canned Soup
Pasta Dinners	Frozen Breakfast Sandwiches, Biscuits & Meals
Frozen Patties and Burgers	Breakfast Sandwiches, Biscuits & Meals
Flavored Snack Crackers	Sweet Bakery Products
Snacks	Prepared Wraps and Burittos
Popcorn, Peanuts, Seeds & Related Snacks	Flavored Rice Dishes
Pasta by Shape & Type	Frozen Pancakes, Waffles, French Toast & Crepes
Other Meats	Frozen Sausages, Hotdogs & Brats
Poultry, Chicken & Turkey	Frozen Poultry, Chicken & Turkey
Meat/Poultry/Other Animals Unprepared/Unprocessed	Bread
Cooked & Prepared	Frozen Bacon, Sausages & Ribs
Frozen Dinners & Entrees	Meat/Poultry/Other Animals Prepared/Processed
Other Deli	Savoury Bakery Products
Cookies & Biscuits	Vegetarian Frozen Meats
Candy	Processed Cereal Products
Crackers & Biscotti	Canned Seafood
Mexican Dinner Mixes	Prepared/Preserved Foods Variety Packs
Chocolate	Canned Tuna
Wholesome Snacks	Puddings & Custards
Biscuits/Cookies	Canned Meat
Pizza	All Noodles
Fruit Prepared/Processed	Grain Based Products / Meals
Frozen Bread & Dough	Stuffing
Vegetable Based Products / Meals	Fish & Seafood
Pre-Packaged Fruit & Vegetables	Eggs & Egg Substitutes
Bacon, Sausages & Ribs	Dairy/Egg Based Products / Meals
Yogurt/Yogurt Substitutes	French Fries, Potatoes & Onion Rings
Entrees, Sides & Small Meals	Deli Salads
Cereals Products - Ready to Eat (Shelf Stable)	Bread/Bakery Products Variety Packs
Pancakes, Waffles, French Toast & Crepes	Prepared Subs & Sandwiches

Table S3. This table provides the grouped FDC food categories that are used in Model 3 of the case study.

Dairy
Soup
Vegetables
Breakfast
Other Frozen Desserts
Snacks
Grains
Side Dish
Dough Based Products / Meals
Protein Foods
Cooked & Prepared
Frozen Dinners & Entrees
Other Deli
Candy
Mexican Dinner Mixes
Chocolate
Pizza
Fruit
Frozen Bread & Dough
Entrees, Sides & Small Meals
Pizza Mixes & Other Dry Dinners
Prepared Wraps and Burittos
Prepared/Preserved Foods Variety Packs
Deli Salads
Prepared Subs & Sandwiches

Table S4. This table provides the final category groups used with the acceptability constraints in Model 3 and the FDC categories contained in each.

Final Category	Included FDC Categories	Final Category	Included FDC Categories
**Protein Foods**	Frozen Patties and Burgers<br>Other Meats<br>Poultry, Chicken & Turkey<br>Meat/Poultry/Other Animals Unprepared/Unprocessed<br>Bacon, Sausages & Ribs<br>Chili & Stew<br>Canned & Bottled Beans<br>Sausages, Hotdogs & Brats<br>Frozen Fish & Seafood<br>Other Frozen Meats<br>Meat/Poultry/Other Animals Sausages Prepared/Processed<br>Pepperoni, Salami & Cold Cuts<br>Frozen Sausages, Hotdogs & Brats<br>Frozen Poultry, Chicken & Turkey<br>Frozen Bacon, Sausages & Ribs<br>Meat/Poultry/Other Animals Prepared/Processed<br>Vegetarian Frozen Meats<br>Canned Seafood<br>Canned Tuna<br>Canned Meat<br>Fish & Seafood<br>Eggs & Egg Substitutes<br>Dairy/Egg Based Products / Meals	**Grains**	Prepared Pasta & Pizza Sauces<br>Pasta Dinners<br>Pasta by Shape & Type<br>Vegetable and Lentil Mixes<br>Croissants, Sweet Rolls, Muffins & Other Pastries<br>Breads & Buns<br>Rice<br>Pasta/Noodles<br>Sweet Bakery Products<br>Flavored Rice Dishes<br>Bread<br>Savoury Bakery Products<br>All Noodles<br>Grain Based Products / Meals<br>Bread/Bakery Products Variety Packs
**Breakfast**	Cereal<br>Frozen Breakfast Sandwiches, Biscuits & Meals<br>Breakfast Sandwiches, Biscuits & Meals<br>Frozen Pancakes, Waffles, French Toast & Crepes<br>Processed Cereal Products<br>Cereals Products - Ready to Eat (Shelf Stable)<br>Pancakes, Waffles, French Toast & Crepes	**Snacks**	Snack, Energy & Granola Bars<br>Chips, Pretzels & Snacks<br>Cakes, Cupcakes, Snack Cakes<br>Dips & Salsa<br>Other Snacks<br>Lunch Snacks & Combinations<br>Flavored Snack Crackers<br>Snacks<br>Popcorn, Peanuts, Seeds & Related Snacks<br>Cookies & Biscuits<br>Crackers & Biscotti<br>Wholesome Snacks<br>Biscuits/Cookies<br>Puddings & Custards
**Vegetables**	Frozen Vegetables<br>Canned Vegetables<br>Vegetable Based Products / Meals<br>Pre-Packaged Fruit & Vegetables<br>Vegetables Prepared/Processed	**Dairy**	Cheese<br>Ice Cream & Frozen Yogurt<br>Yogurt<br>Yogurt/Yogurt Substitutes
**Soup**	Prepared Soups<br>Canned Condensed Soup<br>Other Soups<br>Canned Soup	**Side Dish**	Frozen Appetizers & Hors D'oeuvres<br>Frozen Prepared Sides<br>Stuffing<br>French Fries, Potatoes & Onion Rings
**Fruit**	Fruit Prepared/Processed<br>Canned Fruit	**Other Frozen Desserts**	Other Frozen Desserts
**Cooked & Prepared**	Cooked & Prepared	**Dough Based Products / Meals**	Dough Based Products / Meals
**Frozen Dinners & Entrees**	Frozen Dinners & Entrees	**Other Deli**	Other Deli
**Candy**	Candy	**Mexican Dinner Mixes**	Mexican Dinner Mixes
**Chocolate**	Chocolate	**Pizza**	Pizza
**Frozen Bread & Dough**	Frozen Bread & Dough	**Entrees, Sides & Small Meals**	Entrees, Sides & Small Meals
**Pizza Mixes & Other Dry Dinners**	Pizza Mixes & Other Dry Dinners	**Prepared Wraps and Burittos**	Prepared Wraps and Burittos
**Prepared/Preserved Foods Variety Packs**	Prepared/Preserved Foods Variety Packs	**Deli Salads**	Deli Salads
**Prepared Subs & Sandwiches**	Prepared Subs & Sandwiches		
